# Chemical Vapor Transformation of Lithium Carbonate during Plasma-Assisted ALD Growth of Lithium Phosphate

**DOI:** 10.1021/acs.jpcc.6c00538

**Published:** 2026-04-01

**Authors:** M. J. Pieters, N. T. Hoogendoorn, C. Van Helvoirt, M. Creatore

**Affiliations:** † Department of Applied Physics and Science Education, Eindhoven University of Technology, P.O. Box 513, Eindhoven 5600 MB, The Netherlands; ‡ Eindhoven Institute of Renewable Energy Systems (EIRES), PO Box 513, Eindhoven 5600 MB, The Netherlands

## Abstract

The research on interface engineering in Li-ion batteries by atomic layer deposition (ALD) is presently shifting from binary metal oxides toward Li-containing oxides, phosphates, and fluorides. In parallel, significant attention is devoted to the development of ALD processes based on volatile Li precursors, for future industrial process upscaling. In this work, we employ the novel Li precursor *Lider*, characterized by a vapor pressure of 1 Torr at 129 °C and Si-free ligands, for the ALD synthesis of lithium phosphate (LiPO). Combining *Lider* with an O_2_ plasma (O_2_*) or O_3_ as a coreactant results in the growth of Li_2_CO_3_ films with growth per cycle values of 0.25 and 0.29 Å, respectively, with differences in film crystallinity and mass density. Then, LiPO is synthesized by combining the O_2_*-based *Lider* process, leading to Li_2_CO_3_, with trimethyl phosphate (TMPO) in the supercycle approach, leading to a growth per supercycle of 0.6 Å and excellent uniformity over an 8 in. wafer. Remarkably, XPS shows that the Li_2.7_PO_3.7_ film does not contain carbonate impurities. The adoption of quadrupole mass spectrometry (QMS) reveals that a chemical vapor transformation reaction occurs between TMPO and the Li_2_CO_3_ surface: similarly to trimethyl aluminum (TMA), TMPO is shown to abstract surface carbonate species as CO_2_. This work demonstrates that this chemical vapor transformation mechanism, so far only investigated for surface cleaning of battery electrodes, can also be utilized in ALD supercycles of Li compounds to obtain films without carbonate impurities.

## Graphical abstract

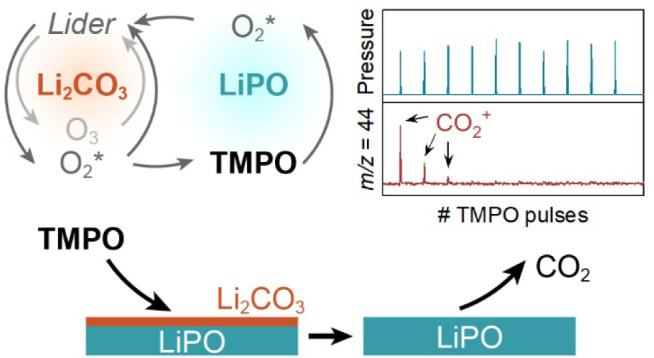

## Introduction

The use of atomic layer deposition (ALD) for interface engineering in Li-ion batteries has been exponentially growing over the past years,^1^ because ALD can deliver conformal film growth on electrodes with complex 3D structures, and it offers good control over film thickness at the Ångstrom level. The relatively low deposition temperature allows ALD processing on temperature-sensitive substrates, such as slurry-cased electrodes that contain polymer binders, or Li metal anodes with their low melting point of 180 °C. Furthermore, ALD can be upscaled and integrated in future battery manufacturing lines, following its implementation in the photovoltaics and nanoelectronics industry.^2,3^ A wide range of battery materials can benefit from ALD layers to improve their interface stability, including cathodes,^4–9^ anodes,^10,11^ and solid electrolytes.^12–14^

So far, mainly ALD binary metal oxides have been used for interface engineering in batteries, with Al_2_O_3_ being the most widely adopted due to the moderate process temperatures and very likely because of the low cost of the trimethylaluminum (TMA) precursor, in view of potential process upscaling.^15,16^ However, there is an increasing interest in ALD of lithium compounds for interface engineering, including lithium-containing oxides,^17–22^ lithium fluorides,^23–27^ and lithium phosphates,^28–31^ because of their wide electrochemical stability window and/or improved ionic conductivity with respect to binary metal oxides. Furthermore, several of these ALD Li compounds have been applied on battery materials, including LiF,^5^ LiP_*x*_O*_y_*,^6^ LiNbO_3_,^32^ Li_3_BO_3_–Li_2_CO_3_,^10^ and LiZr_*x*_O_*y*_.^20^ Besides interface engineering, Li-containing thin films are also of interest for microbattery applications: the ALD fabrication of Li_4_Ti_5_O_12_ anodes,^33^ LiCoO_2_ and LiMn_2_O_4_ cathodes,^34,35^ and LiPON solid electrolytes^36,37^ has been reported.

Battery materials are nonconventional substrates for ALD growth and their complex surface chemistries can result in different growth behavior compared to conventional crystalline Si wafer.^38^ For instance, Li-containing electrode materials generally have a poorly controlled native passivation layer of lithium carbonates and hydroxides, often referred to as “residual lithium”.^39^ Therefore, studies on the effect of ALD layers on these materials can benefit from a surface treatment to clean the electrode surface of residual lithium prior to ALD film growth to ensure consistent results. Plasma exposure has been shown to be effective,^40^ but also exposure to a single gas phase (ALD) reactant can abstract residual lithium species through a chemical vapor transformation mechanism, which has been demonstrated for TMA and HF.^41–43^ This mechanism, so far only investigated for surface cleaning, can also be exploited to abstract carbonate impurities from Li-containing films during ALD growth and it is subject of investigation in the present work.

In parallel to the development of ALD processes and expanding the ALD material library, the ALD community also exploits the design and synthesis of new ALD precursors.^44–47^ Besides properties such as reactivity and thermal stability, volatility and cost are equally relevant to consider, in view of the industrial upscaling of ALD processes. Lithium tert-butoxide (LiO^t^Bu), although widely adopted for the synthesis of ternary materials with low levels of impurities, has low volatility.^18,21,34,48^ Lithium hexamethyldisilazide (LiHMDS) is sufficiently volatile (see Table S1), but the combination of a Si-containing ligand and O_2_ plasma (O_2_*) or O_3_ as coreactant results in the incorporation of Si in the layer,^28,49–51^ which is not always desired and therefore limits its applicability. Furthermore, caution is required when using LiO^t^Bu or LiHMDS in thermal ALD processes: both readily react with H_2_O, which can result in uncontrolled growth.^49,52^ Considering the relevance of ALD processes of Li compounds for the battery industry, the development of new Li precursors is essential, and the research field is actively looking for volatile candidates.^45^

In this work, we adopt *Lider*, which combines high volatility with Si-free ligands. Recently, Zhan et al. reported on use of*Lider* to synthesize lithium phosphate (LiPO) layers on a Mn-based cathode.^53^ To our knowledge, the present work is the first reporting on the development of *Lider*-based ALD processes. First, O_2_* and O_3_-based binary ALD processes are developed and the differences in material properties of the grown Li_2_CO_3_ layers are investigated. Then, the O_2_*-based *Lider* process is combined with trimethyl phosphate (TMPO) in a supercycle to synthesize LiPO, a material that is of interest for interface engineering in Li-ion batteries. Remarkably, despite the presence of the Li_2_CO_3_ subcycle in the LiPO supercycle, the LiPO films are free of carbonate impurities, and we investigate the reason for their absence. We hypothesize that a chemical transformation mechanism takes place where TMPO exposure leads to the abstraction of carbonate species, similarly to what is shown in literature in the case of residual lithium species on battery electrodes when exposed to TMA vapors.^41,43^ To test this hypothesis, we employ quadrupole mass spectrometry (QMS) to monitor the conversion of carbonate surface species to CO_2_ when a Li_2_CO_3_ surface is exposed to TMPO, as well as TMA. Finally, we propose an LiPO ALD reaction mechanism to explain the absence of carbonate impurities in the LiPO films.

## Experimental Details

### ALD Depositions

All ALD depositions were carried out using a commercial FlexAL MK1 ALD reactor (Oxford Instruments) equipped with rotary and turbo molecular pumps that allow for base pressure in the order of 10^–6^ Torr. The reactor is equipped with a remote inductively coupled plasma (ICP) source and an O_3_ generator (Absolute Ozone). The pump unit and the ICP source are connected to the deposition chamber through gate valves. When the table temperature was set below 120 °C, the temperature of the reactor walls was set to the same temperature as the table. For depositions at higher table temperature, the wall temperature was maintained at its maximum of 120 °C. The loadlock of the FlexAL reactor was customized to accommodate the transfer of samples from the loadlock to a N_2_ glovebox using a home-built vacuum suitcase (see Figure S1). For all depositions, Si (100) wafers (Siegert Wafer, 10–20 Ohm·cm) were used as substrates.

*Lider* (Air Liquide), trimethyl phosphate (TMPO, ≥99% Sigma), and trimethyl aluminum (TMA, Dockweiler Chemicals GmbH) were used as precursors and were kept in stainless steel bubblers at 85 °C, 70 °C, and 35 °C, respectively. *Lider* was observed to be stable over time: it was mounted on the ALD reactor and kept at 85 °C for ∼6 months without observing changes in film growth or film properties that could be attributed to precursor degradation. The physical properties of the *Lider* precursor are reported in Section B of the Supporting Information (SI). The *Lider*, TMPO, and TMA supply lines were heated to 120 °C, 90 °C, and 50 °C, respectively, to prevent precursor condensation. TMPO and TMA were dosed into the reactor in vapor drawn mode, while *Lider* was dosed using an Ar carrier gas. A filter with 20 μm pores was placed on the inlet of the *Lider* bubbler to prevent the precursor from entering the Ar line.

During the O_2_ plasma (O_2_*) steps a 50 sccm O_2_ flow rate through the ICP source, a plasma power of 200 W and a chamber pressure of 30 mTorr were used. Before turning on the plasma rf generator, a 1 s pressure stabilization step was used to increase the chamber pressure to 30 mTorr.

When O_3_ was used as coreactant, the O_3_ generator was on during the full ALD process. During the O_3_ dose step, the O_3_ flow was led to the ALD reactor, and the chamber pressure was increased to 900 mTorr. During all other steps of the ALD cycle the O_3_ flow was directed to an O_3_ destructor.

### Characterization Techniques

The film thickness and GPC were determined using *in situ* spectroscopic ellipsometry (SE). A J.A. Woollam, Inc. M2000U tool was used and the obtained data was fitted in the wavelength range 1.2–4.7 eV. The uniformity on 8 in. wafer was measured with *ex situ* SE, using a J.A. Woollam, Inc. M2000X+NIR tool. The data was fitted in the wavelength range 0.7–4.6 eV. The Cauchy formula was adopted for all films.

X-ray photoelectron spectroscopy (XPS) was performed using a Thermo Fisher NEXSA system with a monochromatic Al K-alpha source with a spot size of 400 μm. The base pressure of the system is 10^–9^ mbar. Selected samples were transferred from the N_2_ glovebox to the XPS machine using a vacuum transfer module from Thermo Fisher. For surface scans a pass energy of 50 eV was used. The dual-beam flood gun with low-energy electrons (<1 eV) was used for charge compensation during surface scans on Li_2_CO_3_ films. During surface scans of LiPO films the flood gun was not used. All spectra were calibrated by setting the adventitious C peak in the C 1s spectrum to 284.8 eV. During depth profile measurements on both materials were performed by sputtering with 500 eV Ar ions. The flood gun was on and the “snap” function, with an increased pass energy of 150 eV, was used for faster measurements. The film stoichiometries were determined by applying Avantage Smart backgrounds. The etch time was converted to depth in the depth profile graphs using the film thickness obtained by *in situ* SE, when possible.

A Bruker D8 DISCOVER system (Cu Kα, λ = 1.54060 Å) was used for X-ray diffraction (XRD) and X-ray reflectometry (XRR) measurements to gain information on the film crystallinity and density, respectively. XRD measurements were done in gonio-mode using a step size of 0.02° and an omega offset of 3° to avoid signal from the Si substrate. During the XRR measurements the X-ray intensity were measured as a function of 2Θ between 0° and 6°. The data were analyzed using the Bruker LEPTOS software, and modeled as a Si substrate, a native SiO_2_ interlayer, and layer consisting of the Li-based film.

QMS measurements were performed with a Pfeiffer Vacuum mass spectrometry with a mass-to-charge (*m*/*z*) range of 200 atomic mass units (amu), which is connected to the ALD reactor through a pipeline and a 150 μm diameter pinhole. The system is equipped with a channeltron detector, and the electron energy in the ionizer was set to 70 eV. The pressure in the QMS was kept below 10^–5^ mbar by differential pumping with a turbomolecular pump. During the time-resolved measurements selected *m*/*z* values were tracked using a dwell time of 50 ms. A maximum of 4 *m*/*z* values were measured in one experiment to achieve a time resolution of ∼200 ms. The experiments with TMA and TMPO pulses were repeated several times to verify their reproducibility. Time-resolved QMS measurements during the standard recipe of the *Lider* + O_2_* process and half-cycles were performed as described in Section D of the SI.

## Results and Discussion

First an ALD process based on the *Lider* precursor is developed. In view of synthesizing LiPO, the compatibility of *Lider* with several oxidative coreactants is investigated. Remarkably, within the pressure range in our reactor (1–100 mTorr) we do not observe growth when *Lider* was combined with H_2_O as coreactant. This illustrates that *Lider* is less reactive than LiO^t^Bu and LiHMDS, which do grow thermally under the same conditions.^49,54^ Employing O_2_* or O_3_ as coreactant does result in film growth, and the next section discusses the development of ALD processes based on these two coreactants.

### Li_2_CO_3_ Process Development and Characterization

Saturation curves for the *Lider* + O_2_* process at 150 °C are shown in Figure 1. *Lider* does not react with O_2_ gas, and therefore the O_2_ gas flows during the whole ALD cycle. Figure 1a shows that a *Lider* dose time of 10 s is sufficient to reach saturation. The O_2_* saturation curve in Figure 1b shows an initial decrease in GPC with increasing plasma time, and then saturation behavior. Possible explanations for this trend could be incomplete ligand removal at short plasma times, or film densification due to ion impinging on the film (sub)­surface for longer plasma times.^55^ However, XPS and XRR do not show significant differences in film composition (Table S3) and mass density (Table S4), respectively. Instead, we consider that the plasma gas residence time of 0.7 s is sufficiently long to have redeposition of reaction-product fragments, resulting in an increased GPC, as also reported by Knoops et al.^56^ The selected ALD parameters, shown in Figure S4a, result in a GPC of 0.25 Å. A comparison among other ALD processes based on several Li precursors and oxidants (Table S2) shows that the GPC of the *Lider* + O_2_* process is lower than the one reported for LiO^t^Bu-based Li_2_CO_3_ processes (0.6–0.8 Å),^54^ but close to the GPC of 0.3 Å reported for other Li_2_CO_3_ ALD processes, based on *ex situ* methods.^52,57^ The selected ALD parameters result in a good uniformity: the *Lider* + O_2_* process shows a low nonuniformity of 2.3% on an 8 in. wafer (Figure S5a).

**Figure 1:**
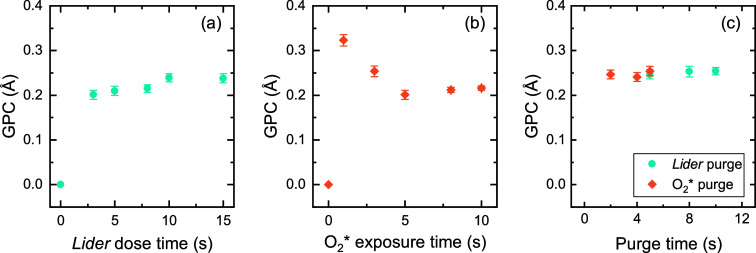
Saturation curves of *Lider* + O_2_* process at 150 °C. Variation of (a) the *Lider* dose time, (b) the O_2_* exposure time, and (c) the purge times, with respect to the initial process parameters: 10 s *Lider* +5 s purge +5 s O_2_* + 5 s purge.

The temperature-dependence of the GPC of the *Lider* + O_2_* process is investigated, with a focus on the low table temperature range, because of the intended application of *Lider*-based ALD processes on (temperature-sensitive) battery electrodes. Consequently, we stay well below the decomposition temperature of *Lider* at >300 °C. Figure 2 shows that the GPC of the *Lider* + O_2_* process decreases with increasing deposition temperature. This trend could be due to a decrease in functional surface groups, e.g., OH-groups, for *Lider* to chemisorb on. However, the investigated temperature range of 100 °C is too narrow to solely attribute the strong decrease in GPC of ∼50% to a decrease in functional groups. For example, in the case of the TMA + O_2_* process, we found that the GPC decreases by ∼10% over the same temperature range (Figure S11).

**Figure 2:**
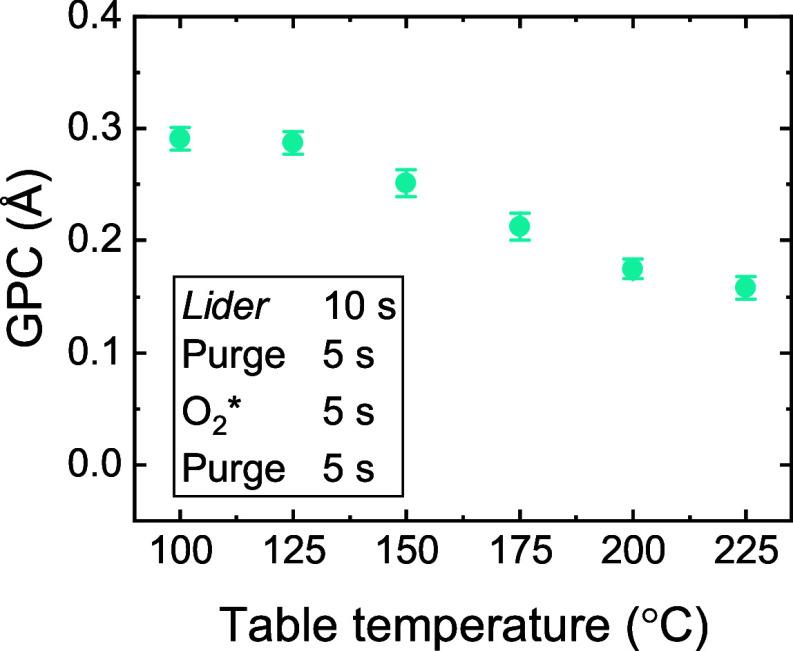
GPC of *Lider* + O_2_* process as a function of deposition temperature.

Instead of dissociative chemisorption, accompanied by ligand abstraction when reacting with surface groups, we argue that film growth is driven by a physisorption-like interaction of *Lider* with the film surface, with the molecule remaining intact. Since we observe saturation behavior for *Lider* and the constant GPC in Figure 1c suggests that there is no precursor desorption during purges, the “physisorption” must be self-limiting and stronger than van der Waals interactions, likely similar to the proposed dipole-driven physisorption process in the case of LiHMDS.^28,49^ Werbrouck et al. first described this physisorption mechanism involving dipole–dipole interactions between the dipole moments in the Li–N bond of the LiHMDS molecule and in O–Li bonds at the film surface. Since the adsorbed molecules screen the surface dipoles and therefore prevent further physisorption, the precursor shows saturation behavior, which is why this mechanism is described as *self-saturated dipole-driven physisorption*.^28^ In the rest of this work the “physisorption” of the *Lider* molecule refers to the mechanism described here.

The hypothesis on the physisorption of *Lider* is supported by time-resolved QMS data (Figure S6a,b): the signal of *m*/*z* = 15 amu, to be attributed to the presence of CH_*x*_ ligands from the precursor, is present during the O_2_* step the standard recipe, together with the signals of combustion products (*m*/*z* = 18, H_2_O^+^; *m*/*z* = 44, CO_2_^+^). Furthermore, the signal of *m*/*z* = 15 amu is absent during the precursor dose, since there is no difference in the signals of the standard recipe and the *Lider*-only half-cycle in Figure S6a. These observations indicate that *Lider* physisorbs during the precursor dose step without losing its ligands, which are subsequently abstracted and combusted by the O_2_*. Higher deposition temperatures would promote precursor desorption from the surface, which would then support the observed trend in Figure 2.

The material properties of the films grown with the *Lider* + O_2_* process at 150 °C are further investigated. The films do not change appearance upon air exposure, which is a first indication that the films do not contain air-sensitive LiOH and/or Li_2_O.^49^ XPS measurements were performed on a film that was air-exposed, and on a film that was transferred from the ALD reactor to the XPS machine under inert conditions using vacuum suitcases. XPS surface scans of both films show features in the O 1s, C 1s and Li 1s spectra that are attributed to Li_2_CO_3_ (Figure 3). Additionally, an XPS depth profile of a film capped with ∼10 nm Al_2_O_3_ (100 cycles TMA + O_2_*) to protect the film against air exposure shows the presence of C in the film underneath the capping layer (Figure S7a). Both results indicate that the *Lider* + O_2_* process results in the ALD growth of Li_2_CO_3_, similar to the LiO^t^Bu + O_2_* process,^54^ and exclude that Li_2_CO_3_ is formed when the ALD film consisting of LiOH and/or Li_2_O reacts with H_2_O and CO_2_ upon air exposure. In line with Hornsveld et al.,^54^ the conversion of LiOH to Li_2_CO_3_ during film growth is attributed to a reaction with CO_2_, that is formed during the O_2_* step:

1 $${\mathrm{2 LiOH}\;(s)+\mathrm{CO}}_{2\;}\left(g\right)\to {\mathrm{Li}}_{2}{\mathrm{CO}}_{3\;}{(s)+H}_{2}O\;\left(g\right)$$

**Figure 3:**
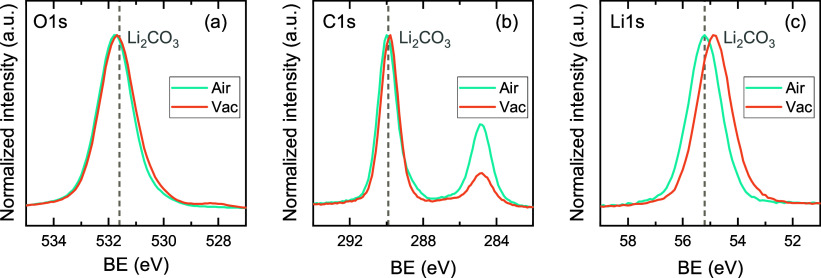
Normalized XPS surface scans of (a) O 1s, (b) C 1s, and (c) Li 1s for films grown with the *Lider* + O_2_* process at 150 °C. The films were either exposed to air before the XPS measurement (Air), or transported between the ALD reactor and XPS equipment using a vacuum suitcase to prevent air exposure (Vac).

However, the XPS results suggest that this reaction does not convert all Li_2_O/LiOH to Li_2_CO_3_ during film growth. The O 1s spectrum of the vacuum-transferred film in Figure 3a shows a second, smaller feature around 528 eV (see Figure S8), which is close to the expected binding energy (BE) of Li_2_O (528.5 eV), similar to the observations of Kozen et al. for the LiO^t^Bu + O_2_* process.^58^ The absence of the Li_2_O feature in the measurement on the air-exposed film, and the shift of the Li 1s feature to higher BE (Figure 3c) show that air exposure completes the conversion of Li_2_O to Li_2_CO_3_.

The crystallinity of the films was investigated by XRD. The diffractogram in Figure 4 shows a diffraction peak at 31.7° that corresponds to the (002) orientation of Li_2_CO_3_. This (002) peak was also observed for other ALD-grown Li_2_CO_3_ films.^52,54,57^ The Li_2_CO_3_ film density measured with XRR (see Table S4 and Figure S10a) is close to the bulk density of Li_2_CO_3_ (2.1 g/cm^3^), and comparable to the densities reported for other ALD-grown Li_2_CO_3_ films.^54^ Also, XRR shows an increase in film thickness of several nm compared to the thickness measured by *in situ* SE prior to air exposure, and significant surface roughness. This is attributed to the chemical conversion of Li_2_O to Li_2_CO_3_ upon air exposure, which was observed by XPS. The adsorption of CO_2_ leads to volume expansion of the layer, which is supported by the increase in film thickness and surface roughness.

**Figure 4:**
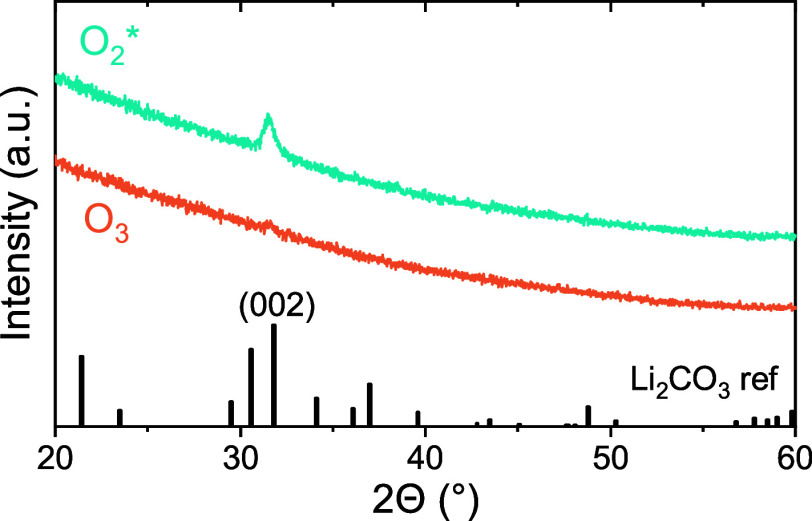
Gonio XRD measurements on a 20 nm film grown with O_2_* as coreactant (blue trace) and a 35 nm film grown with O_3_ as coreactant (orange trace).

In view of exploring the compatibility of various oxidative coreactants with *Lider* as precursor, also O_3_ is investigated as coreactant and found to be sufficiently reactive to result in film growth. The *Lider* + O_3_ ALD process at 150 °C shows saturation behavior for both the precursor and O_3_ dose times, as shown in Figure 5a,b. The GPC of 0.29 Å is higher than the GPC of 0.25 Å for the O_2_*-based process. Figure 5c shows that a relatively long purge time is required after the O_3_ dose step, most likely because the high pressure during the O_3_ dose step results in a longer time needed to remove species from the reactor chamber. The final process parameters, determined from the saturation curves, are shown in Figure S4b, and result in a nonuniformity of 7.4% on an 8 in. wafer (Figure S5b). Similar to the O_2_*-based process, the GPC of the *Lider* + O_3_ process decreases with increasing deposition temperature (Figure 6).

**Figure 5:**
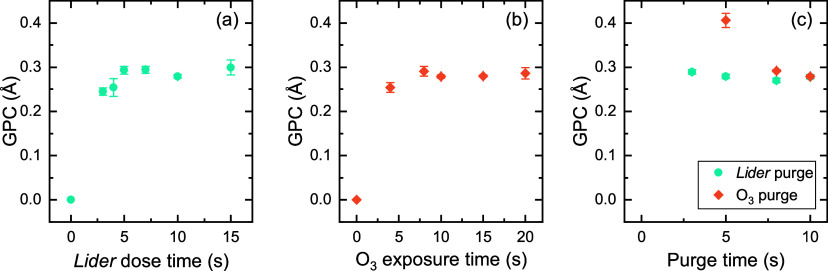
Saturation curves of *Lider* + O_3_ process at 150 °C. Variation of (a) the *Lider* dose time, (b) the O_3_ exposure time, and (c) the purge times, with respect to the initial process parameters: 10 s *Lider* +5 s purge +10 s O_3_ + 10 s purge.

**Figure 6:**
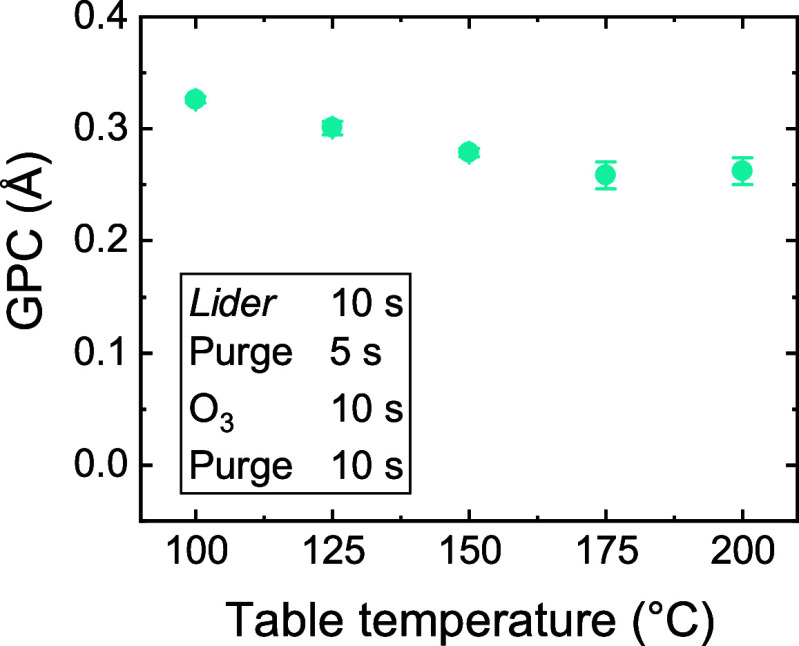
GPC of *Lider* + O_3_ process as a function of deposition temperature.

XPS surface scans in Figure 7 and the depth profile in Figure S7b show that the *Lider* + O_3_ process also results in Li_2_CO_3_ films. However, in contrast to the O_2_*-based process, the vacuum-transferred film shows no Li_2_O feature in the O 1s spectrum. Furthermore, XRD shows no diffraction peaks for the films grown with O_3_ as coreactant (Figure 4), and XRR gives a lower film density (1.8–1.9 g/cm^3^) and surface roughness compared to the O_2_*-based process (Table S4 and Figure S10b). The films did not increase in thickness upon air exposure, in line with the previous conclusion that the film does not consist of air-sensitive Li_2_O.

**Figure 7:**
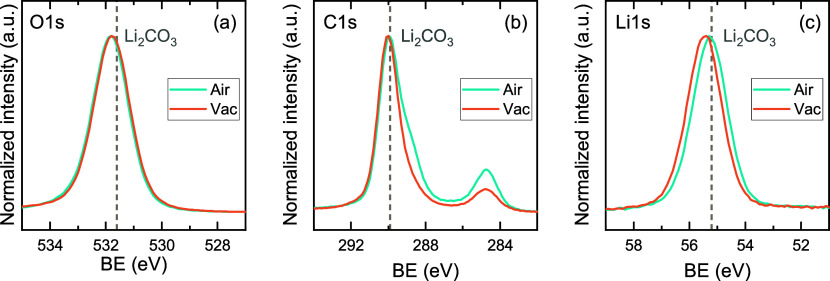
Normalized XPS surface scans of (a) O 1s, (b) C 1s, and (c) Li 1s for films grown with the *Lider* + O_3_ process. The films were either exposed to air before the XPS measurement (Air), or transported between the ALD reactor and XPS equipment using a vacuum suitcase to prevent air exposure (Vac).

The reduced crystallinity and low mass density of the films grown with O_3_ as coreactant could be explained by the absence of energetic ions, that can play a role in densification and crystallization in the O_2_*-based process. However, the (002) diffraction peak observed for thermal ALD Li_2_CO_3_ films grown at 150 °C shows that energetic ions are not required to obtain (partially) crystalline Li_2_CO_3_ films at this deposition temperature.^54^ Another explanation for the difference in crystallinity and density could be the incorporation of a small amount of persistent *Lider* ligands for the O_3_-based process. Figure S9 namely shows that there remains a second feature in the C 1s spectrum at lower BE than the carbonate-related feature after 120 etch seconds. This low BE contribution corresponds to ∼1 at. % and it is attributed to the presence of carbon species from *Lider* ligands incorporated in the film. The absence of this feature in the film grown by the O_2_*-based process indicates that O_2_* is more effective than O_3_ in removing the *Lider* ligands.

To summarize, O_2_* and O_3_ were both investigated as coreactants in a *Lider*-based ALD process. Both lead to the growth of Li_2_CO_3_ films, but O_2_* results in films without C impurities from incorporated ligand fragments, and a higher mass density, and enhanced crystallinity, compared to O_3_. These variations in material properties between the two ALD processes could lead to different outcomes in, for example, electrochemical performance when these layers would be adopted for interface engineering in batteries.

### LiPO Process Development and Characterization

Compared to the O_3_-based process, the *Lider* + O_2_* process results in films with less ligand impurities and a shorter cycle time. Therefore, O_2_* is selected as coreactant to synthesize LiPO. Saturation curves for the *Lider* + O_2_* + TMPO + O_2_* process at 150 °C are shown in Figure 8. Both *Lider* and TMPO dose times show saturation behavior. While the GPC remains constant as the O_2_* step after the *Lider* dose is varied, the duration of the second plasma step after the TMPO dose significantly affects the GPC (Figure 8d). With the selected dose times, shown in the schematic in Figure S12, the LiPO process has a GPC of 0.6 Å, which remains roughly constant in the 100–200 °C temperature range (Figure 9). The GPC is relatively low compared to the GPCs reported in literature for LiPO ALD processes based on LiO^t^Bu (0.7–1.0 Å ^30,31^ ) and LiHMDS (0.4–1.3 Å^29^). However, the GPCs reported in literature correspond to higher deposition temperatures (225–350 °C) and were shown to be strongly temperature-dependent. Furthermore, the GPC of the *Lider* + O_2_* process is low compared to, for example, the LiO^t^Bu + O_2_* process (0.8 Å^54^), which might also contribute to the relatively low GPC of the *Lider*-based LiPO process.

**Figure 8:**
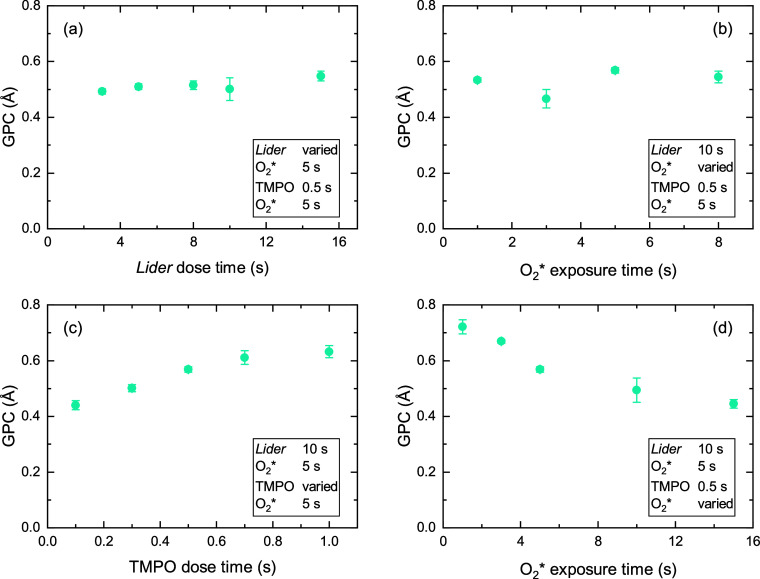
Saturation curves of LiPO supercycle process at 150 °C.

**Figure 9:**
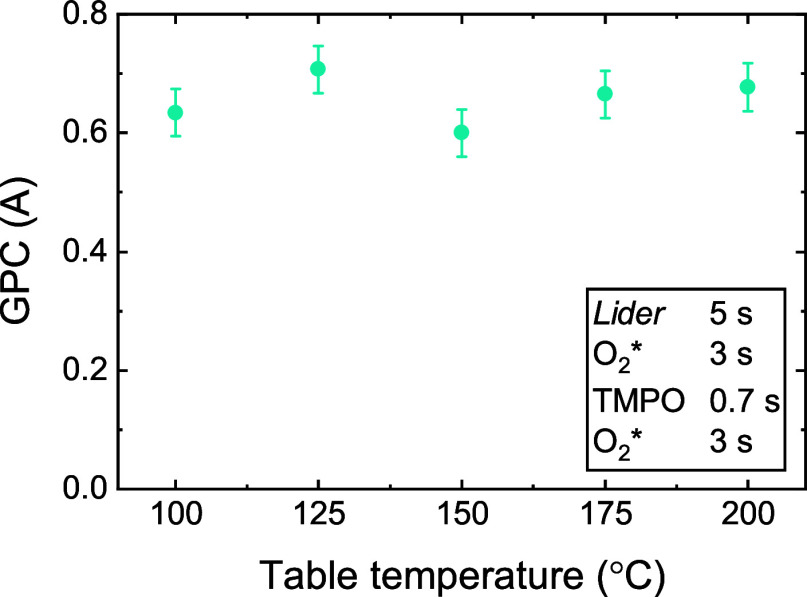
GPC of the LiPO supercycle process as a function of deposition temperature.

Initially a short *Lider* dose time of 5 s was selected, based on the saturation curve in Figure 8a, but this resulted in a high nonuniformity of 35.8% (Figure S13a). XPS showed that the Li at.% was lower on the edge of the wafer compared to the center (Figure S14), suggesting that 5 s *Lider* dose is insufficient to obtain a good uniformity. The uniformity was strongly improved by increasing the *Lider* dose time to 10 s, resulting in a nonuniformity of 0.9% (Figure S13b). Therefore, a 10 s *Lider* dose time was selected for the LiPO supercycle recipe, as shown in Figure S12, and the material properties of the resulting film are further investigated.

XRD shows no diffraction peaks (Figure S15), suggesting that the LiPO film has an amorphous structure. The LiPO film stoichiometry determined from XPS surface scans in Figure 10 is 47.4 ± 0.5% O, 34.4 ± 0.8% Li, 12.8 ± 0.2% P, 5.3 ± 0.2% C, resulting in a film composition of Li_2.7_PO_3.7_, which is close to Li_3_PO_4_. The O 1s spectrum (Figure 10a) has two contributions, which are attributed to bridging oxygen (P–O–P) and nonbridging oxygen (Li–O–P) environments.^31,59,60^ Remarkably, the C 1s spectrum in Figure 10d shows only adventitious C species and no sign of carbonates around 290 eV, even though their incorporation would be expected based on the *Lider* + O_2_* subcycle, that was discussed in the previous section. Also the XPS depth profile (Figure S16) shows that the level of C impurities in the bulk of the LiPO film is below the XPS detection limit. Similarly, Tsuruoka et al. used an O_2_* as coreactant in their LiPO process, which should result in ligand combustion and therefore carbonate formation, but HAXPES showed only ∼1 at. % C.^31^ The mechanism behind the absence of carbonate impurities is here further investigated.

**Figure 10:**
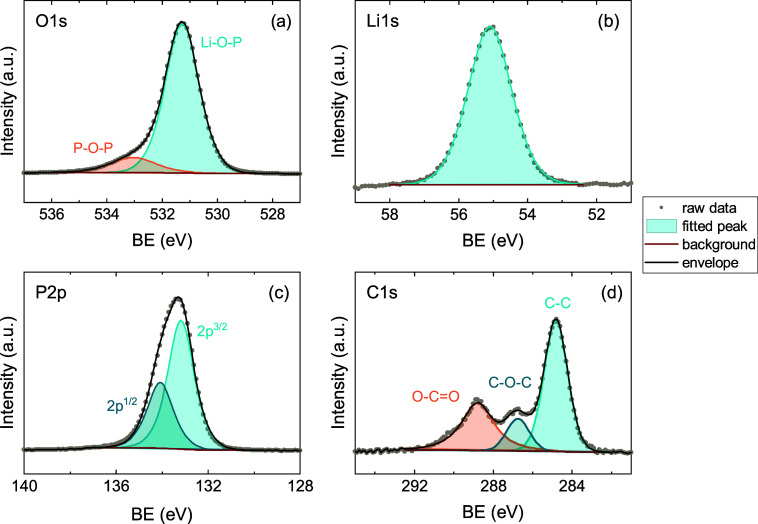
XPS surface scans of (a) O 1s, (b) Li 1s, (c) P 2p and (d) C 1s for a LiPO film grown with the *Lider* + O_2_* + TMPO + O_2_* recipe at 150 °C.

### Carbonate Abstraction during LiPO Film Growth

To explain the absence of carbonate impurities in the LiPO films, we were inspired by studies on the removal of the residual lithium (Li_2_CO_3_) surface layer formed on battery electrode materials upon air exposure. Besides wet-chemical methods based on, for example, a (NH_4_)_2_HPO_4_ solution,^61–63^ also a gas phase approach based on TMA has been investigated.^41,43,64^ Young et al. exposed a LiMn_2_O_4_ cathode to TMA and observed that the Li_2_CO_3_ feature in the C 1s XPS spectrum of the cathode material decreased.^41^ Combining this observation with DFT calculations, they propose that the carbonate removal occurs through a chemical vapor transformation mechanism, where TMA reacts with Li_2_CO_3_, resulting in the formation of CO_2_, CH_4_ and C_2_H_6_. The same group also demonstrated recently that the same chemical vapor transformation mechanism occurs when Li metal anodes with residual Li surface species are exposed to TMA vapors.^43^ Studies that employ QMS to detect gaseous reaction products when TMA is dosed on battery electrodes (with residual lithium surface layers) only report the detection of CH_4_ (*m*/*z* = 15,16 amu) and C_2_H_6_ (*m*/*z* = 28, 29, 30 amu) as reaction products,^4,43,65^ but the abstraction of carbonates in the form of CO_2_ (*m*/*z* = 44 amu) has not yet been monitored. Therefore, in this work we investigate the chemical vapor transformation mechanism between TMA and a Li_2_CO_3_ surface using QMS, and we extend the study to the TMPO molecule in the presence of Li_2_CO_3_. Based on the absence of carbonate impurities in the LiPO films, we hypothesize that TMPO reacts with Li_2_CO_3_ that is formed during the O_2_* steps in the LiPO process, similarly to TMA.

Time-resolved QMS experiments were performed to detect the formation of gaseous reaction products when TMA and TMPO are dosed on a Li_2_CO_3_ surface. Specifically, we focus on the detection of CO_2_, since its formation would indicate that carbonate impurities are abstracted during the TMPO dose in the LiPO supercycle. The QMS signal primarily originates from reactions occurring at the reactor walls (kept at 120 °C) due to their large surface area. Therefore, the reactor walls were first conditioned with Li_2_CO_3_ by running 400 cycles of the *Lider* + O_2_* ALD process, which serves as a model system for the carbonate surface that is expected to form during the O_2_* steps in the LiPO supercycle. Subsequently, 10 pulses of either TMA (30 ms) or TMPO (500 ms) were dosed into the reactor, separated by pump steps. During these precursor pulses, selected *m*/*z* values were measured by QMS. *M/z* = 44 amu is selected to measure the presence of CO_2_. *M/z* = 15 amu corresponds to CH_3_^+^, but this signal can originate from the CH_4_ reaction product, as well as the CH_3_ ligands of the TMA and TMPO precursors. *M/z* = 26 amu (C_2_H_2_^+^) was selected to detect the presence of the C_2_H_6_ reaction product, since this *m*/*z* value does not occur in the mass spectra of CO_2_, TMA and TMPO.^66^

First the reactions between TMA and the Li_2_CO_3_ surface are investigated to verify the chemical vapor transformation mechanism proposed by Young et al.^41^ Figure 11 shows time-resolved QMS measurements and the reactor pressure during the 10 TMA pulses. During the first 1–2 TMA pulses peaks in the *m*/*z* = 44 amu signal are observed, showing that CO_2_ is formed upon TMA exposure. The small signals observed during subsequent pulses are attributed to pressure effects, and not the formation of CO_2_. This suggests that TMA can only react with the Li_2_CO_3_ surface and not deeper in the film, most certainly because of the steric hindrance offered by the chemisorbed TMA molecules. During the first TMA pulse there is no peak visible at *m*/*z* = 57 amu, while the pressure readout in Figure 11d does reveal that TMA is dosed. This indicates that TMA molecules in the gas phase are consumed by chemisorption on the Li_2_CO_3_ surface already during the first pulse. Additionally, a repetition of the same experiment (Figure S17) shows a strong signal of *m*/*z* = 15 amu during the first TMA pulse, which therefore must correspond to the formation of CH_4_ when TMA chemisorbs on polar surface groups. Also, the C_2_H_6_ reaction product is observed at *m/z* = 26 amu, but only from the second TMA pulse onward. This is in line with the observation of Li et al. that C_2_H_6_ is not formed during the first TMA dose on Li_2_CO_3_ and LiOH surfaces.^4^

**Figure 11:**
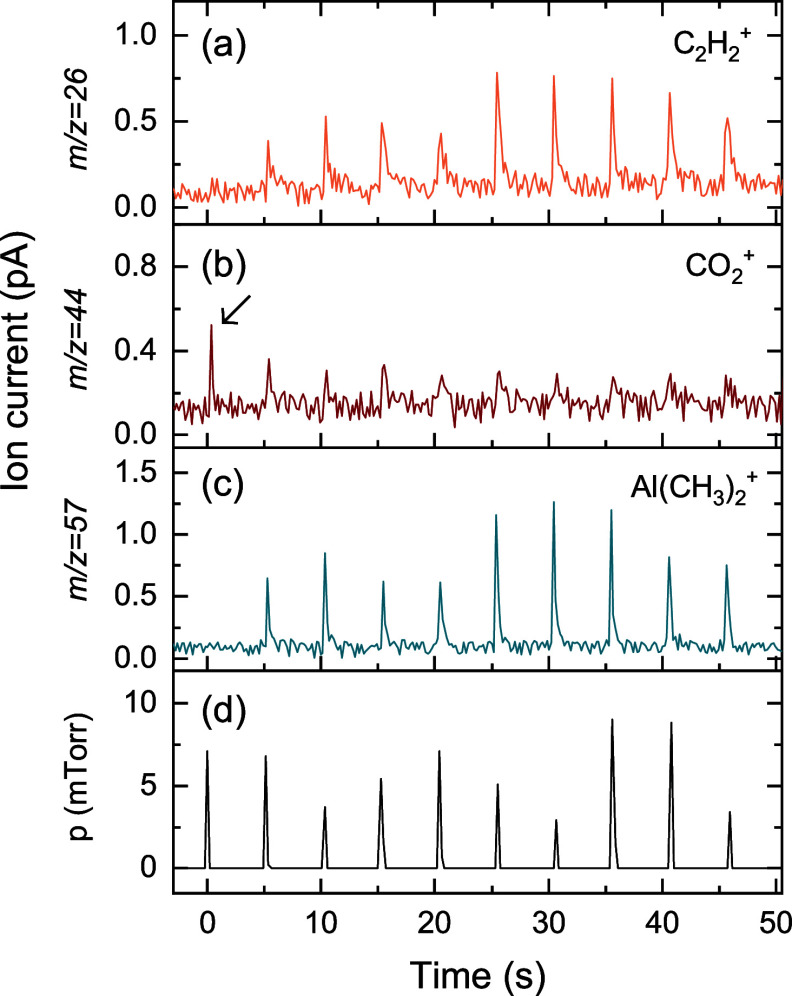
Time-resolved QMS measurements of 10 × [30 ms TMA dose +5 s pump] in a Li_2_CO_3_-conditioned ALD chamber. The QMS signals of (a) *m*/*z* = 26, (b) 44, and (c) 57 amu are attributed to the presence of C_2_H_6_, CO_2_, and TMA, respectively. (d) Pressure in the reactor. The fluctuation in peak height is attributed to the low time resolution (∼125 ms) of the pressure readout with respect to the TMA dose time (30 ms).

Parallel experiments were performed to demonstrate that TMPO also promotes the conversion of carbonate groups into CO_2_, as shown in Figure 12. The presence of the signal at *m*/*z* = 44 amu (Figure 12c) during the first ∼3 TMPO pulses indicates the formation of CO_2_. Besides CO_2_, TMPO and TMA pulses on a Li_2_CO_3_ surface result in different reaction products. The constant intensity of the *m*/*z* = 15 amu signal (Figure 12a), upon multiple TMPO doses shows that CH_4_ is not a reaction product of TMPO chemisorption, in line with the observation of Hornsveld et al.^67^ Furthermore, no C_2_H_6_ signal was observed (*m*/*z* = 26 amu, Figure S18), indicating that the C_2_H_6_ reaction product is unique to TMA. Instead, the peaks at *m*/*z* = 31 amu in Figure 12b follow the same trend as the CO_2_ peaks, which suggests that a reaction product with this mass is formed during the first ∼3 TMPO pulses. Kozen et al. propose methanol (CH_3_OH) and formaldehyde (CH_2_O) as possible reaction products during the TMPO dose.^59^ Since formaldehyde, with a parent ion of *m*/*z* = 30 amu, cannot explain the signal at *m*/*z* = 31 amu, the signal is here attributed to the formation of methanol.

**Figure 12:**
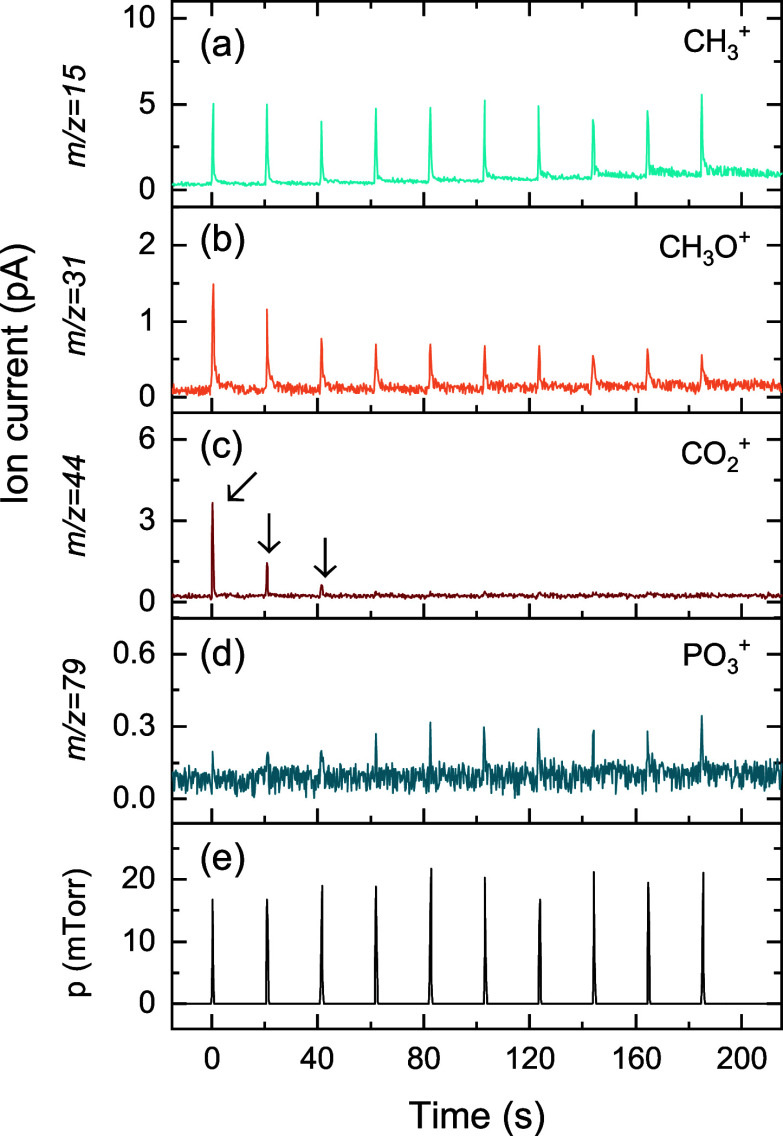
Time-resolved QMS measurements of 10 × [500 ms TMPO dose + 20 s pump] in a Li_2_CO_3_-conditioned ALD chamber. The QMS signals of (a) *m*/*z* = 15, (b) 31, (c) 44, and (d) 79 amu are attributed to the presence of CH_4_/TMPO, CH_3_OH/TMPO, CO_2_ and TMPO, respectively. (e) Pressure in the reactor.

The *m/z* = 79 amu signal, which is indicative of TMPO, remains low during the first 3 TMPO pulses and then increases and stays constant from the fourth pulse onward. This indicates that TMPO is removed from the vapor phase by reacting with the Li_2_CO_3_ surface during the first 3 pulses. From the fourth pulse TMPO no longer reacts with the surface and is more widely present in the vapor phase. This is in line with the observed saturation behavior of TMPO in the LiPO ALD process in Figure 8c.

Based on the XPS and QMS results a reaction mechanism is proposed for the LiPO film growth in Figure 13. First, *Lider* physisorbs via dipole–dipole interactions, as discussed earlier in this work, and the subsequent O_2_* step combusts the *Lider* ligands, based on the detection of CO_2_ and H_2_O as byproducts. Part of the surface OH-groups react with the formed CO_2_, converting LiOH to Li_2_CO_3_, in accordance with eq 1. When this mixed carbonate- and hydroxyl-terminated surface is exposed to TMPO vapors, we argue that the following reactions occur. TMPO chemisorbs on surface OH-groups, accompanied by the loss of CH_3_OH. Additionally, TMPO displaces the carbonate groups, resulting in chemisorbed TMPO and the release of CH_3_OH and CO_2_. The carbonate removal and formation of Li_3_PO_4_ could be justified by the difference in the standard Gibbs free energy of formation (ΔG_f_^o^) between Li_3_PO_4_ (−1970 kJ/mol) and Li_2_CO_3_ (−1132 kJ/mol).^68,69^ Moreover, part of the adsorbed TMPO molecules form P–O–P bonds, as confirmed by XPS (Figure 10d). The bridging O is presumably formed during the TMPO dose step due to polymerization between TMPO molecules, since the table temperature is below the ceiling temperature (∼300 °C^70^). The final O_2_* step combusts the methyl ligands of the chemisorbed (Me)_*x*_PO, creating a surface for the *Lider* precursor to physisorb onto in the subsequent step.

**Figure 13:**
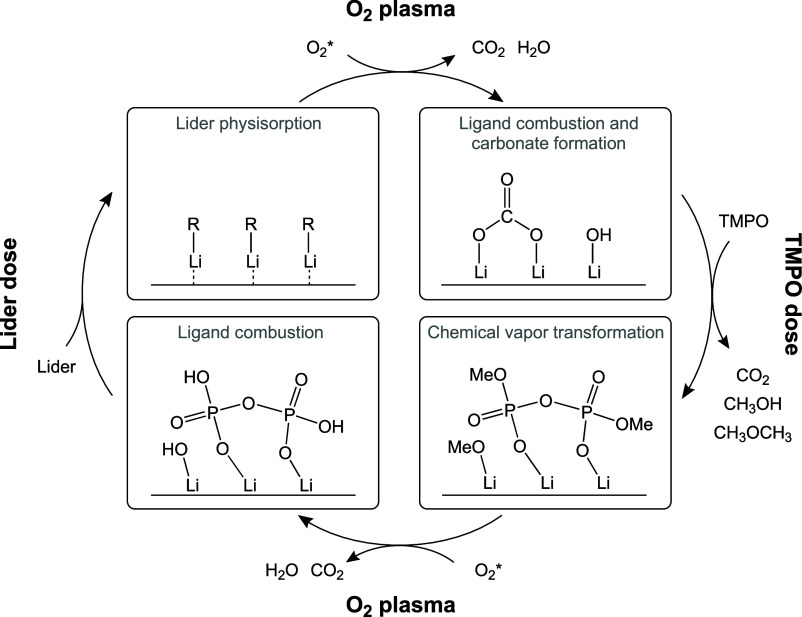
Schematic of proposed reaction mechanisms of LiPO film growth based on QMS studies.

## Conclusion

In this work Li_2_CO_3_ and LiPO ALD processes were developed based on the novel Li precursor *Lider*, which is characterized by good volatility and Si-free ligands. Combining *Lider* with O_2_* and O_3_ as coreactants resulted in uniform Li_2_CO_3_ films with GPCs of 0.25 and 0.29 Å, respectively, at 150 °C. XPS revealed that ∼1 at. % of C in the O_3_-based Li_2_CO_3_ films originates from incorporated *Lider* ligands, which presumably contributes to the amorphous nature and relatively low mass density (1.8–1.9 g/cm^3^) of the O_3_-based Li_2_CO_3_ films. In contrast, the O_2_*-based Li_2_CO_3_ films do not contain impurities, have a higher mass density of 2.0–2.1 g/cm^3^, and are (partially) crystalline.

The *Lider* + O_2_* process was applied in a supercycle with TMPO for the synthesis of LiPO. The *Lider* + O_2_* + TMPO + O_2_* supercycle showed a GPSC of 0.6 Å at 150 °C, and a low nonuniformity of <1% on an 8 in. wafer. LiPO films can be grown at low substrate temperatures (100–150 °C), which allows processing on e.g., Li metal anodes. XPS showed that the LiPO film has a Li_2.7_PO_3.7_ stoichiometry, and is free of carbonate impurities, despite the Li_2_CO_3_ subcycle in the LiPO supercycle.

Upon the chemical vapor transformation reaction between TMA and surface Li_2_CO_3_ on battery electrodes reported in literature, it was hypothesized that TMPO abstracts carbonates from the LiPO film during film growth. Therefore, time-resolved QMS was used to monitor the reaction products when TMA or TMPO are dosed on a Li_2_CO_3_ surface. It was found that carbonate surface species are removed as CO_2_ during the first few precursor pulses, which was attributed as the reason for the absence of carbonate impurities in the LiPO films. Besides CO_2_, the TMA doses resulted in CH_4_ and C_2_H_6_ as reaction products, in line with literature, and CH_3_OH was measured as reaction product in the case of TMPO. Whereas this chemical vapor transformation reaction was already known for TMA, it has not yet been reported for TMPO.

The formation of carbonates during ALD synthesis of Li compounds is associated with the adoption of O_2_* and O_3_ coreactants, and it is therefore not unique to *Lider*-based ALD processes. This work shows that these surface carbonates can be abstracted during film growth by specific ALD precursors: TMA and TMPO. However, the driving force behind the chemical vapor transformation mechanism is not yet fully understood, and therefore carbonate abstraction should not be assumed to occur for other ALD precursors. Further research on understanding the driving force, for example based on computational methods, could aid in identifying other precursor chemistries that exhibit a similar reactivity toward Li_2_CO_3_, and could therefore be employed in an O_2_* or O_3_-based ALD supercycle to synthesize other ternary Li compounds that are free of unwanted carbonate impurities.

## Supplementary Material



## Data Availability

The data underlying this study are openly available in the 4TU. ResearchData repository at https://doi.org/10.4121/bb89726d-b52c-4cbd-aa88-a573a47719a6.

## Notes

Additional information on the homebuilt vacuum suitcase, physical properties of the *Lider* precursor, a literature overview of ALD processes based on Li precursors and oxidizing coreactants, schematics of ALD process parameters, uniformity measurements, XRR data and fitting, additional XPS, XRD, and QMS data (PDF)
