# Supplementary material for: Chemical Vapor Transformation of Lithium Carbonate during Plasma-Assisted ALD Growth of Lithium Phosphate
Source: J Phys Chem C Nanomater Interfaces. 2026 Apr 1;130(15):5458–69. doi: 10.1021/acs.jpcc.6c00538 (PMC13093485; doi:10.1021/acs.jpcc.6c00538)
Supplement: Supplementary file 1 [file jp6c00538_si_001.pdf]

Supporting information for

Chemical Vapor Transformation of Lithium Carbonate During Plasma-Assisted ALD Growth of Lithium Phosphate

M. J. Pieters[a,*], N. T. Hoogendoorn[a], C. Van Helvoirt[a], M. Creatore[a,b]

[a] Department of Applied Physics and Science Education, Eindhoven University of Technology, P.O. Box 513, 5600 MB Eindhoven, The Netherlands

[b] Eindhoven Institute of Renewable Energy Systems (EIRES), PO Box 513, 5600 MB Eindhoven, The Netherlands

*Electronic mail: m.j.pieters@tue.nl

A. Homebuilt vacuum suitcase

The homebuilt vacuum suitcase consists of an Al carrier plate and a stainless steel lid with an O ring, as shown in Figure S1. When samples are removed from the ALD reactor, the lid is placed on top of the carrier plate in the loadlock, while it is still evacuated. Afterwards, the loadlock is vented, and the pressure difference between in- and outside of the lid creates an air-tight seal. The lid can be removed, e.g. after transport to a glovebox, by opening the vent screw on top.

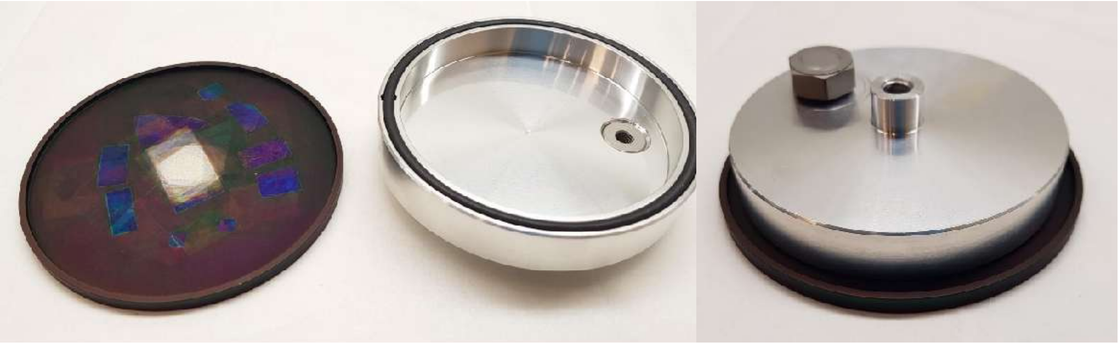

Figure S1 – Photos of the homebuilt vacuum suitcase that is used to transfer samples in vacuum between the ALD reactor and a N_2 glovebox.

B. Physical properties of *Lider* precursor

Lider is an organometallic precursor containing C, H, N, O and Li atoms.

The thermal stability and evaporation temperature of *Lider* were studied by TGA and DTA (Figure S2). The minimum in the heat flow curve around 80°C indicates the melting temperature of *Lider*. The second minimum around 108 °C is attributed to the onset of evaporation. The weight loss occurs in a single step without leaving substantial residues (<1 %) at 300 °C, which indicates that decomposition temperature lies above 300 °C. Measurements of the vapor pressure (Figure S3) yield an evaporation enthalpy ΔH_{vap} of 77 kJ/mol.

Table S1 compares the physical properties of *Lider* with other Li precursors. It should be noted that the final evaporation/sublimation temperature varies depending on loading. The values shown here for LiO^tBu, LiHMDS and Li(thd) are measured using the same loading (10 mg), but the Lider value is measured using a higher loading (35 mg). Therefore these final evaporation/sublimation temperatures should not be directly compared to each other.

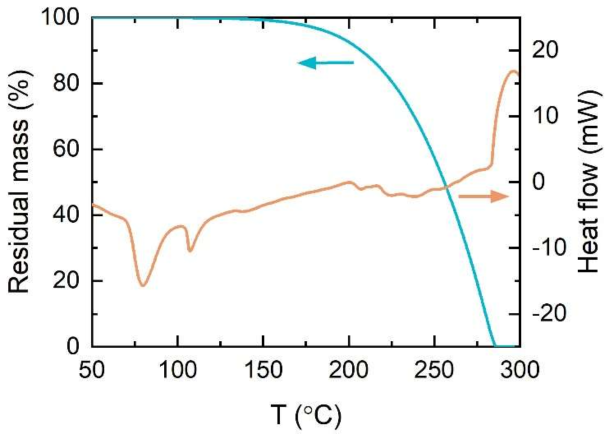

Figure S2 – TGA and DTA curves of Lider precursor at atmospheric pressure (760 Torr), as provided by Air Liquide.

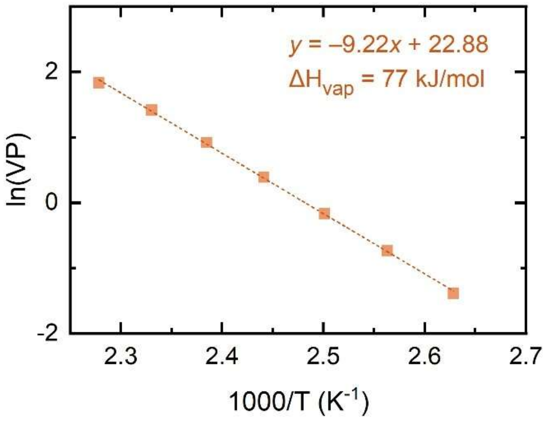

Figure S3 - ln(P) vs 1000/T plot, as provided by Air Liquide. The slope of the linear fit (dashed line) is used to determine ΔH_{vap}.

Table S1 – Comparison of the physical properties of Lider to other available Li precursors. Data is provided by Air Liquide, unless another reference is provided.

Li precursor	Volatility 1Torr (°C)	Melting point (°C)	Final evaporation/sublimation temperature (°C)
**LiO^tBu**	>160[1]	No melting point observed with TGA	302 (10 mg)
**LiHMDS**	~99 (25 mg, step isotherm method)	95	225 (10 mg)
**Li(thd)**	~220-240[2]	No melting point observed with TGA	298 (10 mg)
Lider	129 (25 mg, full isotherm method)	80	285 (35 mg)

C. Literature overview

Table S2 – Overview of ALD processes based on a Li precursor and H_2O, O_2 and/or O_3 as coreactant*

Precursor	Source temperature	Substrate temperature	Coreactant	Grown material	GPC	ref
**LiOtBu**	140°C	100-300 °C<br>50-250 °C<br>275-300 °C	H_2O + CO_2<br>O_2*<br>O_2*	Li_2CO_3<br>Li_2CO_3<br>Li_2O	0.6 Å<br>0.8 Å<br>1.2-1.3 Å	3
	165°C	<240 °C<br>≥240 °C<br>225 °C	H_2O<br>H_2O<br>O_2*	LiOH<br>Li_2O<br>Li_2CO_3/Li_2O	0.1 Å<br>0.1 Å<br>0.4 Å	4
**Li(thd)**	175-200°C	185-225 °C	O_3	Li_2CO_3	0.3 Å	5
**LiTMSO**	165°C	175-300 °C<br>200-300 °C	H_2O + CO_2<br>O_3 + H_2O	Li_2CO_3<br>$LiSi_xO_y$	0.3-0.5 Å<br>1.5 Å	6
**LiHMDS**	85°C	120-250 °C	H_2O<br>O_2*	Li_2O<br>$LiSi_xO_y$	Uncontrolled<br>1.2 Å	7
	90°C	150-300 °C	O_2*	$LiSi_xO_y$	1.4-1.8 Å	8
	75°C	100-330 °C	H_2O<br>H_2O + CO_2	Not reported<br>Li_2CO_3	Uncontrolled<br>0.3 Å	9
	60°C	150-350 °C	O_3	LiSiOx	0.3-1.4 Å	10
Lider	85°C	100-225 °C	H_2O<br>O_2*<br>O_3	-<br>Li_2CO_3<br>Li_2CO_3	No growth<br>0.25 Å<br>0.29 Å	**This work**

D. Li_2CO_3 ALD processes

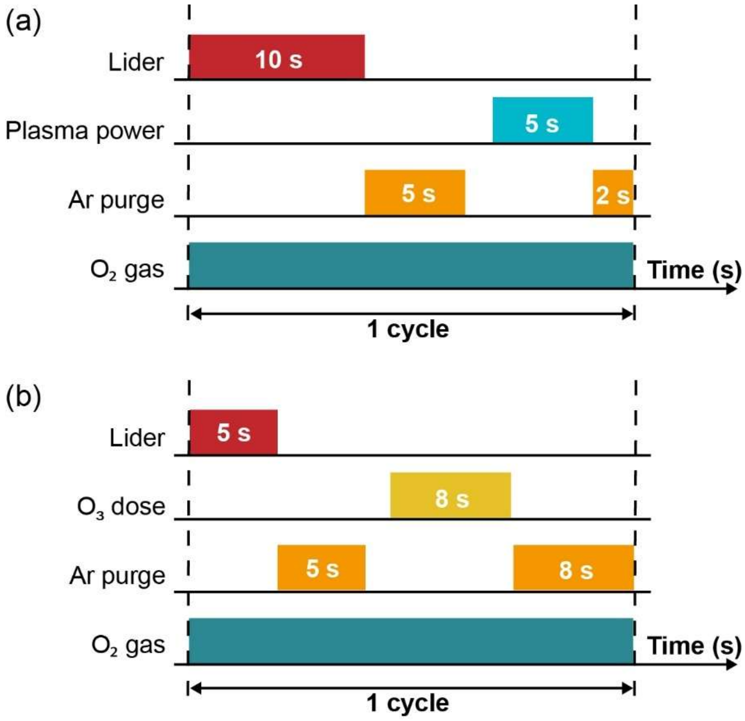

Figure S4 –Schematic of the selected ALD process parameters of (a) the Lider + O_2 process and (b) Lider + O_3 process at 150 °C.*

Table S3 – Composition of Li_2CO_3 films grown with the Lider + O_2 ALD process at 150 °C with varying O_2* dose times, determined from XPS surface scans of air-exposed films. The difference in C at.% can be attributed to variations in adventitious carbon species.*

O_2* dose time (s)	Li at.% (± 0.8%)	C at.% (± 0.5%)	O at.% (± 1.0%)
1	33.4	19.2	47.3
5	34.1	22.5	43.4

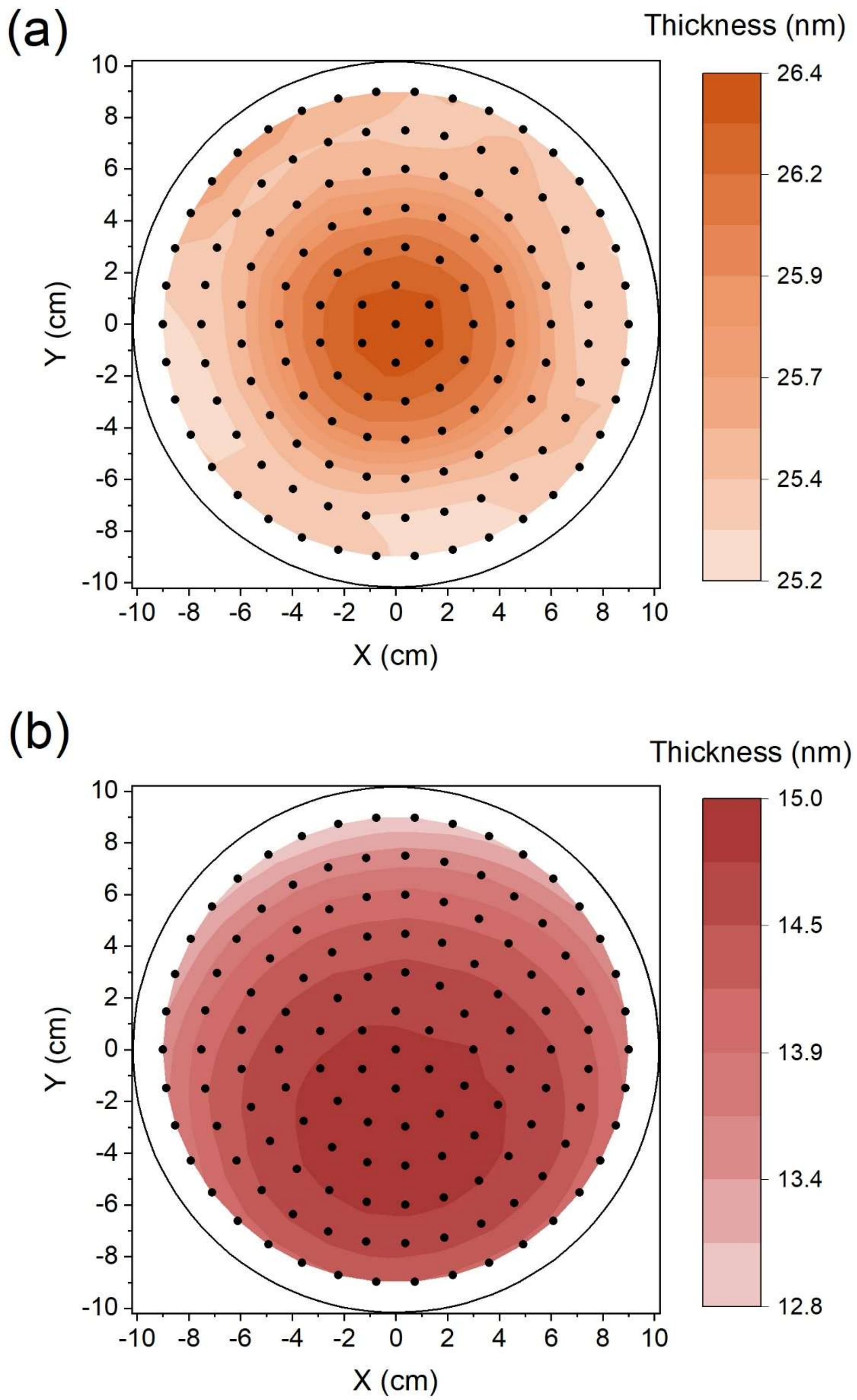

Figure S5 – Uniformity of film thickness (a) Lider + O_2 and (b) Lider + O_3 ALD processes on 8 inch wafers. The wafer size is indicated by the black circle, and the measurement locations are indicated by black points. The optical constants were assumed to be constant. The non-uniformities are (a) 2.3% and (b) 7.4%.*

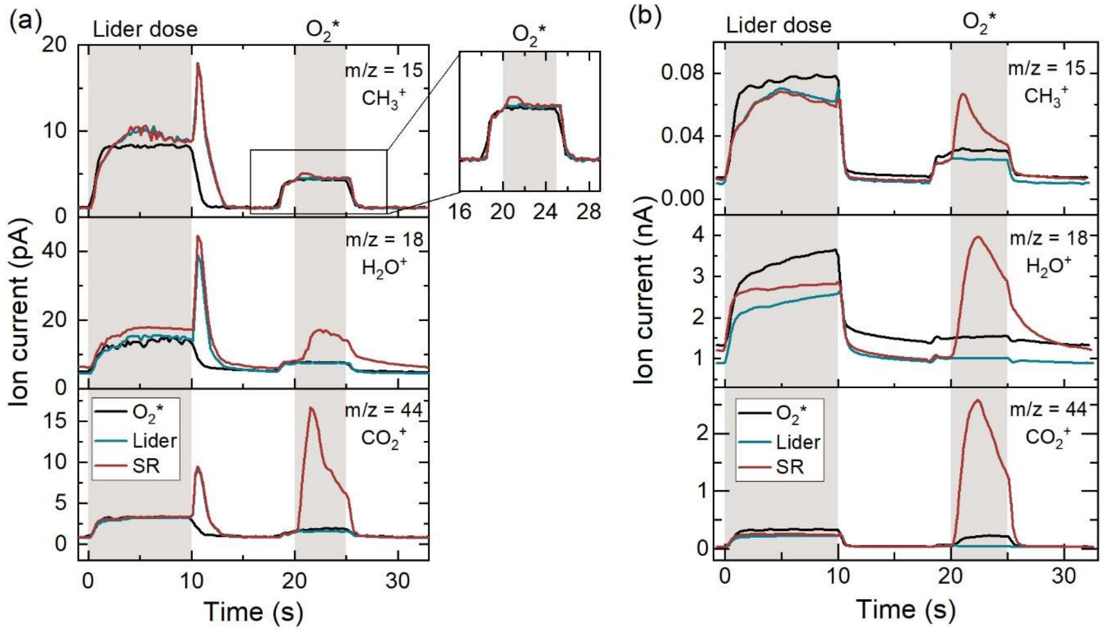

Figure S6 – Time-resolved QMS data of selected m/z values during the Lider + O_2 process. The standard ALD recipe (SR, red) is compared to the half-cycle recipes with only O_2* (black) and only Lider and O_2 gas (blue). (a) includes a zoom of the m/z=15 amu region during the O_2* step. (b) shows a repetition of the same experiment to verify the reproducibility of the m/z=15 amu signal during the O_2* step.*

The time-resolved QMS experiments shown in Figure S6 are performed similarly to those reported in previous work [7]. Selected m/z values were tracked per channel using a dwell time of 50 ms. A maximum of 5 m/z values were measured simultaneously, such that the time resolution was at least 250 ms. Measurements were taken during the standard ALD cycles, and during half-cycles, in which one of the reactants has been left out. This allows to distinguish between background signals (e.g., precursor fragmentation in the QMS, pressure-related signal) and the formation of ALD reaction products. For all processes at least 10 (half) cycles were monitored to verify that a steady state was achieved.

The peaks observed in Figure S6a at the end of the Lider dose for all m/z values for the standard recipe (SR) and the *Lider*-only half-cycle are due to a pressure effect, originating from a filter on the inlet of the *Lider* bubbler. This results in an Ar pressure pulse when switching from dosing *Lider* to purging. During the *Lider* dose step no signs of released

ligands (*m/z* = 15) are observed. During the O_2* step, a small ligand signal is observed, along with combustion products (CO_2, H_2O). This suggests that the *Lider* ligands remain on the surface during the *Lider* dose step, and are abstracted and combusted during the plasma step.

The repeated measurements in Figure S6b show that the m/z=15 amu signal during the O_2* step is reproducible. The differences in the baseline of ion currents during the purge steps, particularly in case of m/z=15 and 18 amu, could be due to pressure differences in the ALD reactor between the various measurements.

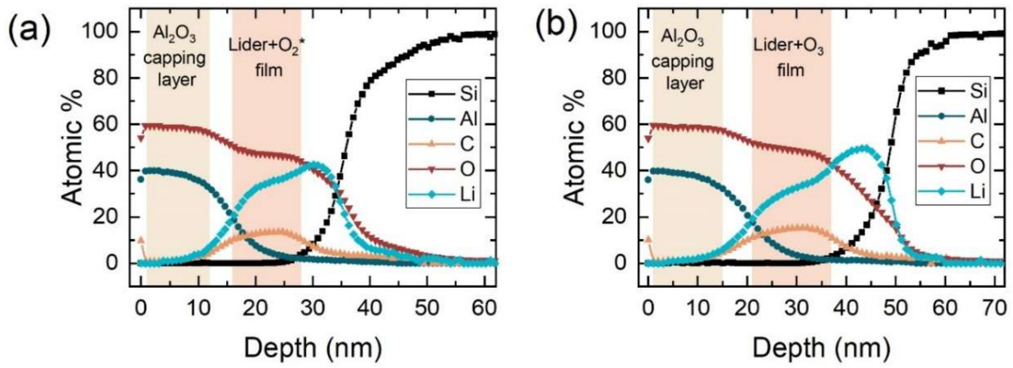

Figure S7 – XPS depth profiles of films grown with the Lider + O_2 (a) and Lider + O_3 (b) ALD processes. The Li-based films were capped with ~10 nm Al_2O_3.*

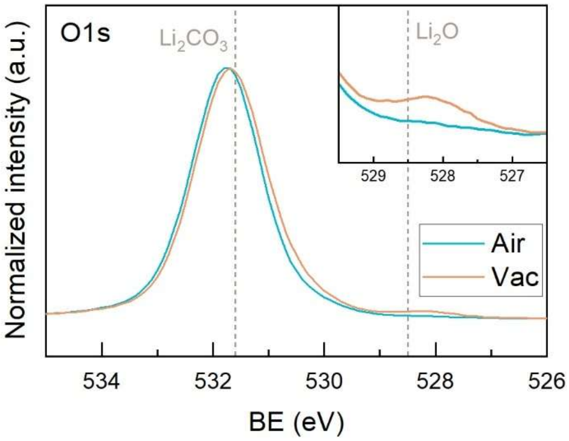

Figure S8 – Normalized O1s XPS surface scans for films grown with the Lider + O_2 process, as shown in Figure 3a. The films were either exposed to air before the XPS measurement (Air), or transported between the ALD reactor and XPS machine using vacuum suitcases to prevent air exposure (Vac). The inset shows a zoom of the low BE region.*

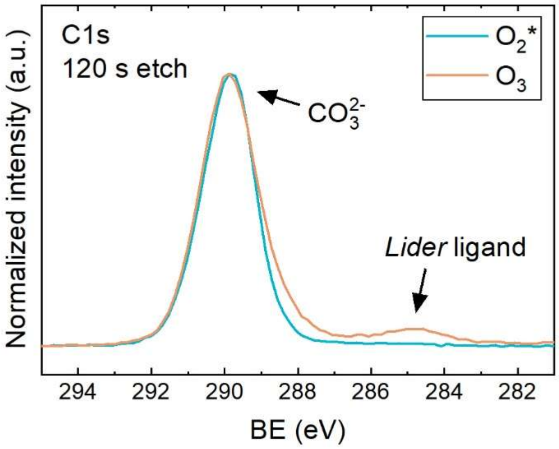

Figure S9 - XPS C1s spectra after 120 etch seconds of Li_2CO_3 films grown with the Lider + O_2 (blue) and Lider + O_3 (orange) ALD processes. Because of the absence of adventitious carbon, the binding energy scales are corrected by setting the carbonate peak to 289.8 eV.*

The raw XRR data was fitted to determine the mass density of the Li_2CO_3 films. The uncertainty in the film density and thickness was determined by fitting the same data over with various fit constraints. The standard deviation of the resulting set of values was taken as the uncertainty, shown in Table S4.

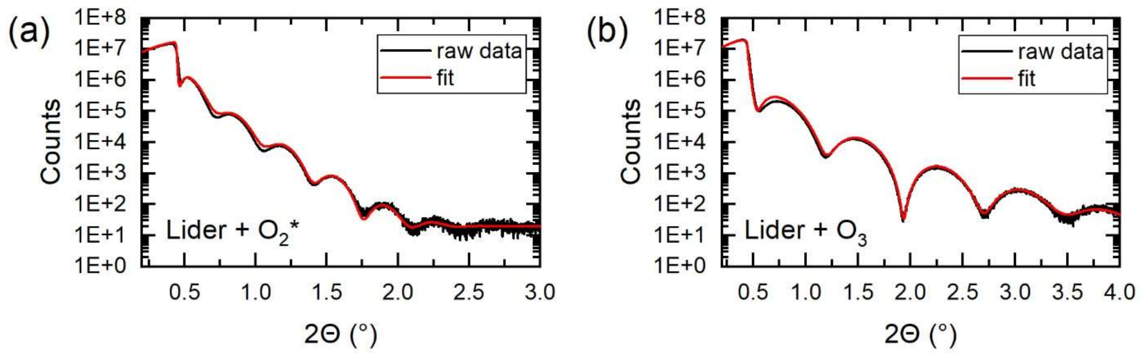

Figure S10 –XRR raw data and an example of a fit used to determine film thickness and mass density of Li_2CO_3 films grown using the Lider + O_2 (a) and Lider + O_3 (b) ALD recipes.*

Table S4 – Film density, thickness, and roughness of Li_2CO_3 films grown using the Lider + O_2 (standard and short O_2* time) and Lider + O_3 ALD recipes determined using XRR, and thickness of the same film measured by in situ SE.*

**ALD process**		**Density XRR (g/cm^3)**	**Thickness XRR (nm)**	**Roughness XRR (nm)**	**Thickness *in situ* SE (nm)**
**Lider + O_2***	Sample 1	2.0 ± 0.1	25 ± 1	2.2 ± 0.2	20.2 ± 0.6
	Sample 2	2.1 ± 0.2	21.7 ± 0.3	1.6 ± 0.2	19.2 ± 0.4
	Sample 3	2.06 ± 0.05	15.5 ± 0.2	2.08 ± 0.05	12 ± 1
**Lider + O_2* (1s plasma)**	Sample 1	2.2 ± 0.1	16.9 ± 0.2	0.8 ± 0.2	15.7 ± 0.8
**Lider + O_3**	Sample 1	1.92 ± 0.06	9.9 ± 0.4	0.7 ± 0.1	10.0 ± 0.8
	Sample 2	1.80 ± 0.02	17.0 ± 0.2	0.8 ± 0.1	17 ± 1

E. TMA + O_2* process

The decrease in GPC for the Lider + O_2* process with increasing table temperature is compared to the decrease in GPC for the TMA + O_2* ALD process on the same ALD reactor and in the same temperature range. The GPC of the TMA + O_2* process decreases with ~10% between table temperatures of 100 °C and 200 °C.

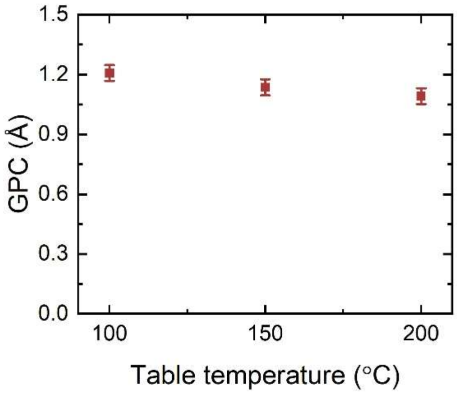

Figure S11 - GPC of TMA + O_2 process as function of deposition temperature*

F. LiPO ALD process

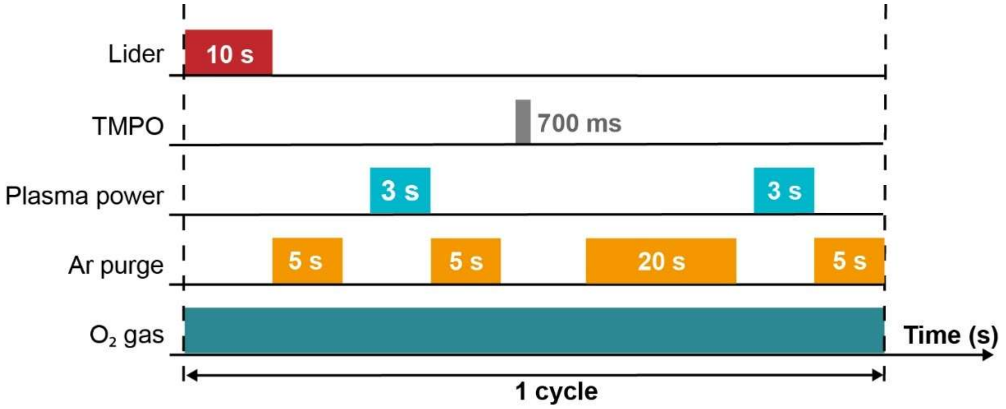

Figure S12 – Schematic of the selected process parameters of LiPO supercycle at 150 °C. After the TMPO dose step, the recipe has a 4 s hold step to give TMPO time to react. A relatively long purge of 20 s after the TMPO dose was used, because shorter purges often resulted in uncontrolled, higher GPSC values, which is attributed to CVD-like contributions because of remaining TMPO in the reactor chamber.

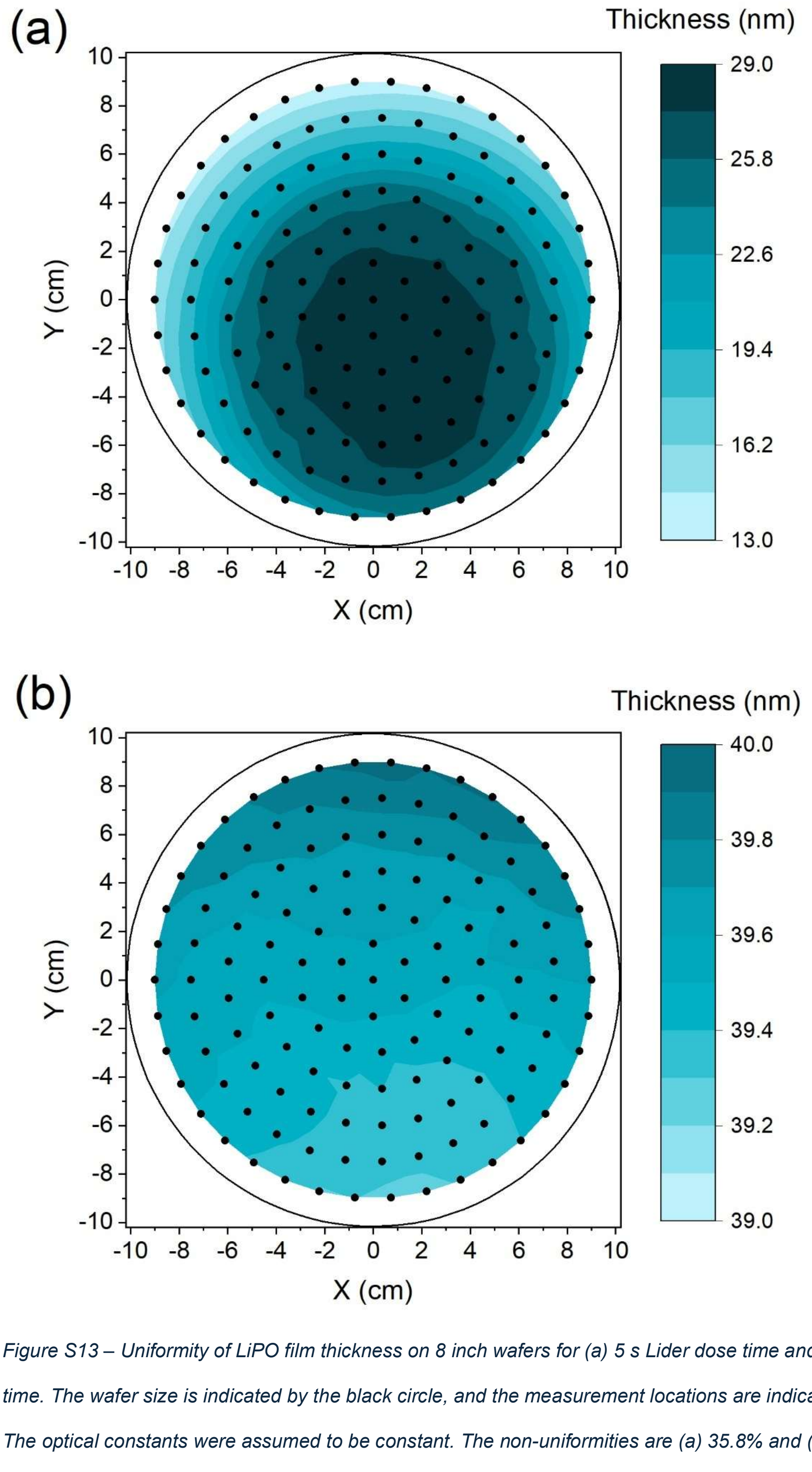

Figure S13 – Uniformity of LiPO film thickness on 8 inch wafers for (a) 5 s Lider dose time and (b) 10 s Lider dose time. The wafer size is indicated by the black circle, and the measurement locations are indicated by black points. The optical constants were assumed to be constant. The non-uniformities are (a) 35.8% and (b) 0.9%.

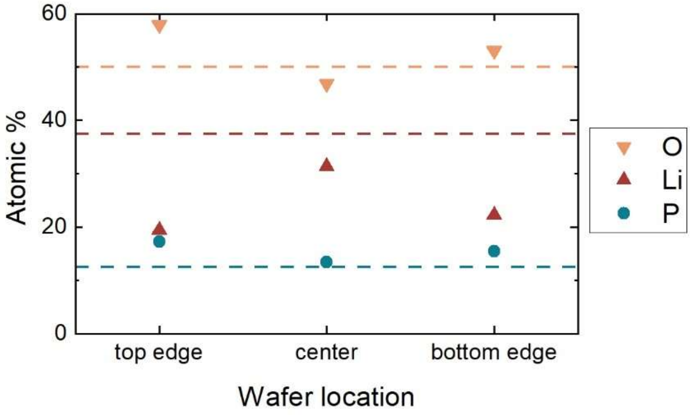

Figure S14 – Li, P and O atomic % determined from XPS surface measurements on different locations on the 8 inch wafer coated with the LiPO ALD process with short Lider dose time. 'Top', 'center' and 'bottom' are with respect to the orientation of the 8 inch wafer as shown in Figure S13a. The horizontal dashed lines represent the Li_3PO_4 stoichiometry.

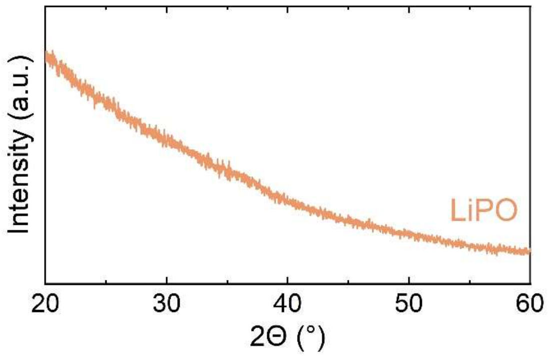

Figure S15 – Gonio XRD measurements on a 20 nm LiPO film.

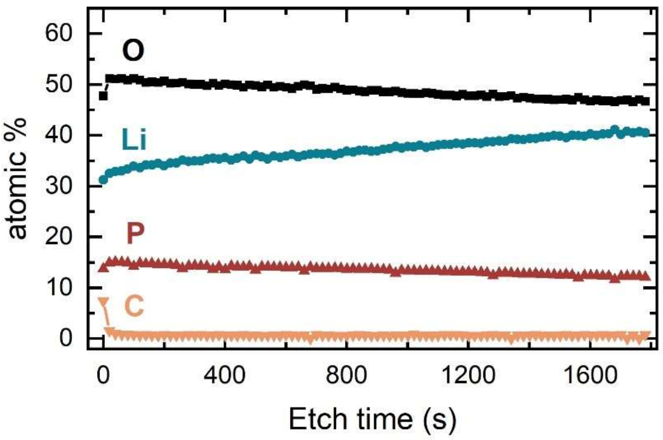

Figure S16 – XPS depth profile of a 40 nm LiPO film. The etch time was not long enough to reach the Si substrate.

G. Additional QMS data for TMA/TMPO pulses

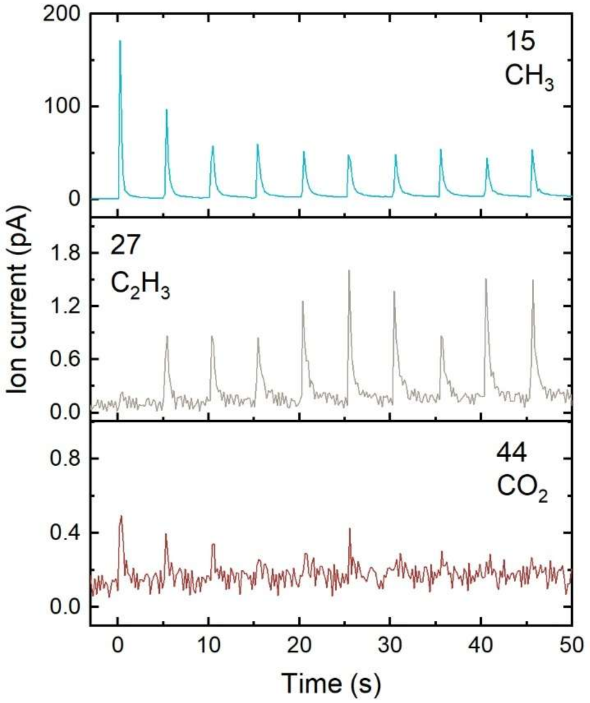

Figure S17 - Time-resolved QMS measurements of 10 x [30 ms TMA dose + 5 s pump] on a Li_2CO_3 surface (150 cycles Lider + O_2). M/z=15, 27 and 44 amu reveal the presence of CH_4 and TMA, C_2H_6 and CO_2, respectively. The time axis is calibrated such that the first TMA pulse starts at t=0 s.*

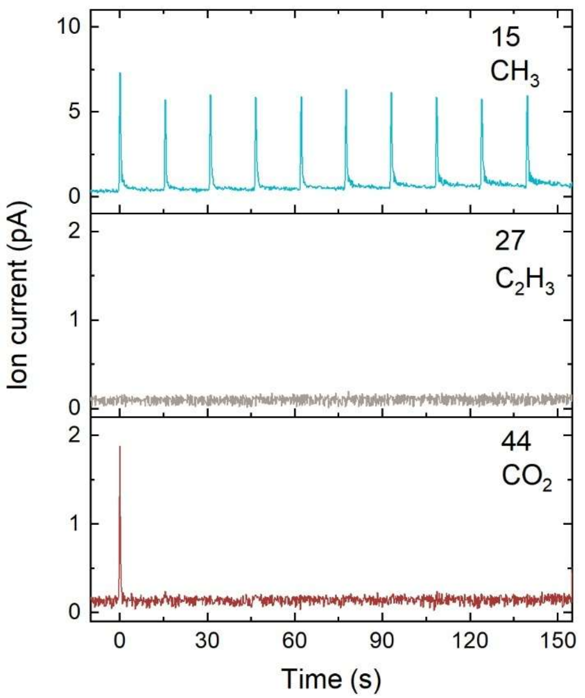

Figure S18 - Time-resolved QMS measurements of 10 x [500 ms TMPO dose + 10 or 20 s pump] on Lider + O_2 film. M/z=15, 27 and 44 measure the presence of CH_4/TMPO, C_2H_6, and CO_2, respectively. The time axis is calibrated such that the first TMPO pulse starts at t=0 s.*

References

(1) Sternschulte, H.; Schreck, M.; Stritzker, B.; Bergmaier, A.; Dollinger, G. Control of Lithium-t-Butoxide Addition during Chemical Vapour Deposition of Li-Doped Diamond Films by Optical Emission Spectroscopy. *Phys. Status Solidi A* **1999**, *174* (1), 65–72.

(2) Filatov, E. S.; Stabnikov, P. A.; Semyannikov, P. P.; Trubin, S. V.; Igumenov, I. K. Synthesis and Thermal Properties of Some Lithium Β-Diketonates. *Russ. J. Coord. Chem.* **2006**, *32* (2), 126–129.

(3) Hornsveld, N.; Put, B.; Kessels, W. M. M.; Vereecken, P. M.; Creatore, M. Plasma-Assisted and Thermal Atomic Layer Deposition of Electrochemically Active Li_2CO_3. *RSC Adv.* **2017**, *7* (66), 41359–41368..

(4) Kozen, A. C.; Pearse, A. J.; Lin, C. F.; Schroeder, M. A.; Noked, M.; Lee, S. B.; Rubloff, G. W. Atomic Layer Deposition and In Situ Characterization of Ultraclean Lithium Oxide and Lithium Hydroxide. *J. Phys. Chem. C* **2014**, *118* (48), 27749–27753.

(5) Putkonen, M.; Aaltonen, T.; Alnes, M.; Sajavaara, T.; Nilsen, O.; Fjellvåg, H. Atomic Layer Deposition of Lithium Containing Thin Films. *J. Mater. Chem.* **2009**, *19* (46), 8767–8771..

(6) Ruud, A.; Miikkulainen, V.; Mizohata, K.; Fjellvåg, H.; Nilsen, O. Enhanced Process and Composition Control for Atomic Layer Deposition with Lithium Trimethylsilanolate. *J. Vac. Sci. Technol. Vac. Surf. Films* **2017**, *35* (1), 01B133.

(7) Pieters, M. J.; Bartel, L.; Van Helvoirt, C.; Creatore, M. Role of the Coreactant on the Dual-Source Behavior of Lithium Hexamethyldisilazide for ALD Li-Containing Films. *J. Phys. Chem. C* **2024**, *128* (46), 19638–19647.

(8) Werbrouck, A.; Mattelaer, F.; Minjauw, M.; Nisula, M.; Julin, J.; Munnik, F.; Dendooven, J.; Detavernier, C. Reaction Pathways for Atomic Layer Deposition with Lithium Hexamethyl Disilazide, Trimethyl Phosphate, and Oxygen Plasma. *J. Phys. Chem. C* **2020**, *124* (50), 27829–27839.

(9) Østreng, E.; Vajeeston, P.; Nilsen, O.; Fjellvåg, H. Atomic Layer Deposition of Lithium Nitride and Carbonate Using Lithium Silylamide. *RSC Adv.* **2012**, *2* (15), 6315–6322.

(10) Hämäläinen, J.; Munnik, F.; Hatanpää, T.; Holopainen, J.; Ritala, M.; Leskelä, M. Study of Amorphous Lithium Silicate Thin Films Grown by Atomic Layer Deposition. *J. Vac. Sci. Technol. A* **2012**, *30* (1), 01A106.
